# MasterPATH: network analysis of functional genomics screening data

**DOI:** 10.1186/s12864-020-07047-2

**Published:** 2020-09-14

**Authors:** Natalia Rubanova, Guillaume Pinna, Jeremie Kropp, Anna Campalans, Juan Pablo Radicella, Anna Polesskaya, Annick Harel-Bellan, Nadya Morozova

**Affiliations:** 1 Institut des Hautes Etudes Scientifiques, Le Bois-Marie 35 rte de Chartres, 91440 Bures-sur-Yvette, France; 2 Université Paris Diderot, Paris, France; 3 Skolkovo Institute of Science and Technology, Skolkovo, Russia; 4 Institute for Integrative Biology of the Cell (I2BC), CEA, CNRS, Univ. Paris-Sud, Université Paris-Saclay, 91198 Gif-sur-Yvette cedex, France; 5 Institute of Molecular and Cellular Radiobiology, Institut François Jacob, CEA, F-92265 Fontenay-aux-Roses, France; 6 INSERM, U967, bâtiment 56 PC 103 18 route du Panorama, BP6 92265 Fontenay-aux-Roses Cedex, France; 7 Université Paris Sud, U967, bâtiment 56 PC 103 18 route du Panorama, BP6 92265 Fontenay-aux-Roses Cedex, France; 8 Ecole Polytechnique, Université Paris-Saclay, CNRS UMR 7654, Laboratoire de Biochimie, Ecole Polytechnique, 91128 Palaiseau, France

**Keywords:** Network analysis, Molecular pathway, Centrality, Loss-of-function screening, Muscle differentiation, DNA repair

## Abstract

**Background:**

Functional genomics employs several experimental approaches to investigate gene functions. High-throughput techniques, such as loss-of-function screening and transcriptome profiling, allow to identify lists of genes potentially involved in biological processes of interest (so called hit list). Several computational methods exist to analyze and interpret such lists, the most widespread of which aim either at investigating of significantly enriched biological processes, or at extracting significantly represented subnetworks.

**Results:**

Here we propose a novel network analysis method and corresponding computational software that employs the shortest path approach and centrality measure to discover members of molecular pathways leading to the studied phenotype, based on functional genomics screening data. The method works on integrated interactomes that consist of both directed and undirected networks – HIPPIE, SIGNOR, SignaLink, TFactS, KEGG, TransmiR, miRTarBase. The method finds nodes and short simple paths with significant high centrality in subnetworks induced by the hit genes and by so-called final implementers – the genes that are involved in molecular events responsible for final phenotypic realization of the biological processes of interest. We present the application of the method to the data from miRNA loss-of-function screen and transcriptome profiling of terminal human muscle differentiation process and to the gene loss-of-function screen exploring the genes that regulates human oxidative DNA damage recognition. The analysis highlighted the possible role of several known myogenesis regulatory miRNAs (miR-1, miR-125b, miR-216a) and their targets (AR, NR3C1, ARRB1, ITSN1, VAV3, TDGF1), as well as linked two major regulatory molecules of skeletal myogenesis, MYOD and SMAD3, to their previously known muscle-related targets (TGFB1, CDC42, CTCF) and also to a number of proteins such as C-KIT that have not been previously studied in the context of muscle differentiation. The analysis also showed the role of the interaction between H3 and SETDB1 proteins for oxidative DNA damage recognition.

**Conclusion:**

The current work provides a systematic methodology to discover members of molecular pathways in integrated networks using functional genomics screening data. It also offers a valuable instrument to explain the appearance of a set of genes, previously not associated with the process of interest, in the hit list of each particular functional genomics screening.

## Background

Functional genomics employs diverse experimental approaches to investigate gene functions. High-throughput techniques, such as loss-of-function screening and transcriptome profiling, allow the identification of specific sets of genes involved in biological processes of interest (so called hit list of genes). Genome-wide loss-of-function screenings exploit gene knock-down or knock-out at the scale of whole genomes. In the context of such screens, RNA interference [1–6], or CRISPR [7–9] libraries are systematically tested in cell-based assays specific to biological function of interest leading to the identification of the regulator genes [10]. Transcriptome profiling aims at profiling mRNA expression levels, e. g. in 2 or more conditions, and at identifying those genes that are up or down regulated. The most widespread techniques for transcriptome profiling are DNA microarrays and RNA-seq techniques [11, 12].

Numerous computational methods for interpretation of functional genomics data sets, inferring molecular machinery underlying a given biological process, have been developed in the past decade, and can be roughly grouped into two categories. The first category encompasses pathways analysis methods, aiming at searching for statistical enrichment of genes with annotated biological process or molecular functions. The classical representatives of the pathways analysis methods are Over Representation Analysis (ORA) methods, which use a statistical test to assess the enrichment of a list of genes in an annotated biological process, molecular function or canonical pathway. The most commonly used statistical tests are based on the hypergeometric, Fisher’s exact, chi-square, or binomial tests [13]. Several improvements of the standard ORA were developed, including functional class scoring approaches that aim at detecting coordinated changes in pathways [13] and topology-based approaches that consider pathway topology, connectivity and interactome information [14, 15].

The second category is network analysis methods which use molecular interaction networks as a supporting information [16]. Such methods can help to find functionally related biological components in a functional genomics data set. This can be achieved in several ways: by introducing network-based scoring methods using e. g. “guilt by association” principle and information from both network topology and screening results [17]; by introducing the use of the connectivity of subgraphs of protein-protein interaction networks [18]; by using network neighbor information [19]; by performing functional analysis that relies on assessing the clustering of selected nodes on the network [20]; by extracting the largest connected component of a subnetwork that is created from the optimal number of the top-ranked genes [21]. Another way to use molecular interaction networks is to find significantly enriched subnetworks within a functional genomics data set. Even manual investigation of such subnetworks can give biologically meaningful results [22, 23]. The molecular interactions networks can be integrated with other types of biological information to achieve higher network specificity: with canonical pathways [24, 25]; with different types of regulatory interactions [25–27]. Moreover, subnetworks can be analyzed for finding functional modules [28, 29].

Here we present a novel network analysis method to analyze functional genomics data sets. The method uses the results of functional screening data to elucidate members of molecular pathways that contribute to the studied phenotype. In contrast to other network analysis methods that work on the level of subnetworks, our method searches for short paths and separate nodes specific to a biological system. Moreover, it shows how hit genes can be associated with these specific paths and nodes. The method works on an integrated interactome (network of molecular interactions) of an organism under investigation. The main theoretical assumption underlying the algorithm is that an observed phenotypic effect of a gene knockdown/knockout, measured as a read-out of a loss-of-function screen, is a sum of the effects of the gene silencing on all molecular pathways influencing the realization of the phenotypic effect. This hypothesis explains the appearance in the hit list of each particular loss-of-function screen of a set of genes, previously not associated with the process under investigation, because a knockdown of each of these genes can trigger several particular molecular pathways, specific for this biological system. On the other hand, the method, built based on this hypothesis, is able to determine a set of the most important pathways in a particular biological system, using the list of hit genes from a genome-wide loss-of-function screen.

According to this theory, the shortest paths from all hit genes to so called final implementers (the genes that are involved in molecular events responsible for final phenotypic realization) are built within the integrated interactome network, and the corresponding subnetwork is extracted.

Next, centrality scores for each node (respective each linear path) in the subnetwork are calculated as the number of the shortest paths that pass through the node (respective the number of the shortest paths for which linear paths are subpaths). Then, the statistical significance of each centrality score is assessed by comparing it with centrality scores in subnetworks built from the shortest paths for randomly generated hit lists preserving the degree distribution of the initial hit list. We hypothesize that the nodes and the linear paths with statistically significant centrality score can be considered as putative members of active molecular pathways leading to the studied phenotype.

The method works with the shortest paths approach to find connections between hit genes and final implementers. It can be expected that this approach can yield incomplete molecular paths especially if biologically meaningful molecular paths are long since current interactomes are known to be incomplete and contain false-positive interactions. In this case, high centrality scores will highlight those segments (nodes and linear paths) that are parts of many shortest paths between different pairs of the hit genes and the final implementers which increases the likelihood that particular segment is specific to the studied phenotype.

Additionally, we demonstrate that being initially created for the analysis of loss-of-function screening results, the method can be well applied for analysis of the results obtained by other high-throughput approaches such as transcriptome profiling. Although, it should be noted that the main drawback of the transcriptome profiling is that this technique does not discriminate the mRNAs that are causal and consequential to the phenotype. Also, we show that if the current knowledge cannot provide the list of final implementers of the process investigated by screening, the program can use a list of hit genes as a list of final implementers, and that putative molecular pathways obtained by this way have good confidence.

We illustrate the application of the method to the analysis of the results of loss-of-function screening and transcriptome profiling of terminal muscle differentiation, and of the results of loss-of-function screening of a DNA repair process.

## Results

### MiRNA loss-of-function screen and transcriptome profiling of human muscle differentiation process

The screening data from the study by A. Polesskaya et al. [30] was taken as the hit list for terminal human skeletal muscle differentiation process. In this study, genome-wide miRNA loss-of-function screening on a late differentiating human muscle precursor cell line (LHCN) was performed in a two-step approach. The primary screening was done in duplicate with a miRNA antisense inhibitors (Locked Nucleic Acids, LNA) library targeting 870 miRNAs and a readout assay that detects Myosin Heavy Chain (MHC) positive and multinucleated myotubes. Those miRNAs whose depletion resulted in differences to the negative control ≥2 standard deviation (SD) were selected for confirmation in the secondary screen. A total of 63 miRNAs (Table S1) whose depletion resulted in differences to the negative control ≥2 SD were confirmed in the secondary screen.

The transcriptome profiling data from the study by J. Kropp et al. [31] was taken as the second hit list for terminal human muscle differentiation process. Transcriptome profiling for proliferation and late differentiation stages in LHCN cell line was performed using Affymetrix Human Gene 1.1 ST arrays [31]. A total of 571 genes (Table S2) were found to be differentially expressed genes with at least 2-fold change between late differentiation and proliferation stage.

As a list of final implementers of the process of human muscle differentiation were taken the proteins responsible for activation, inhibition, facilitating of fusion of myotubes and for the maturation of muscle fibers. Namely, we have selected two major regulatory cytokines that control the muscle size in vivo and in vitro (MSTN and IGF2), three key cytoskeletal proteins that form the contractile apparatus (ACTA1, MYH1, MYLPF), and six plasma membrane-associated proteins (ARF6, CD81, CD9, CDC42, EHD2, MYOF) that have been shown to control the skeletal muscle fusion by a number of different mechanisms [32–39]. Taken together, these final implementers represent key molecular mechanisms of terminal muscle differentiation.

We found 2609 shortest paths of 4 types of length (from 2 to 5 interactions) from each miRNA in the hit list from loss-of-function screening to each protein in the list of final implementers. The subnetwork constructed from these paths consists of 1063 nodes (384 of which are genes) and 2710 edges without duplicated edges. The centrality score and the *p*-value were calculated for each node and path in the subnetwork according the procedure described in the *Methods* section. 521 paths of length of 3 to 4 interactions, with centrality score ≥ 3 and 519 nodes with centrality score ≥ 3 were found at the false discovery rate (FDR) of 0.25. Analysis of the paths with high centrality scores had highlighted a possible role for a number of nuclear receptors (AR, NR3C1) in skeletal muscle differentiation, as well as suggested functions in myogenesis for such proteins as arrestin (ARRB1 and 2), intersectin (ITSN1), the Rho GTP exchange factor VAV3, and the teratocarcinoma-derived growth factor (TDGF1). Interestingly, while the IGF1 regulatory role in myogenesis is very well studied, our approach allowed us to include the arrestin proteins in these pathways, and thus to elaborate the known IGF1 network in skeletal muscle differentiation. The MEF2D, p300, CCND1 functions in differentiation have been abundantly demonstrated, and their presence among the results serves as a proof of efficiency of the analysis.

We found 47,714 shortest paths of 4 types of length (from 1 to 5 interactions) from each gene in the hit list from transcriptome profiling to each protein in the list of final implementers. The subnetwork constructed from these paths consists of 2847 nodes and 13,032 edges without duplicated edges. The centrality score and the *p*-value were calculated for each node and each path in the subnetwork. 905 paths of length of 3 to 4 interactions and centrality score ≥ 3 and 149 nodes with centrality score ≥ 3 were found at the FDR of 0.25. There are 12 miRNAs among these 149 nodes. Three of them (hsa-mir-125b, hsa-mir-133a, hsa-mir-145) are the hit miRNAs in the loss-of-function screen. Five miRNAs with the highest centrality score are hsa-mir-125b, hsa-mir-371, hsa-mir-216a, hsa-mir-1, has-mir-224. These miRNAs, except for hsa-mir-371, were shown to be involved in muscle differentiation and/or proliferation [40–43]. Moreover, almost half of the 12 miRNAs are known to participate in terminal muscle differentiation, and potential roles in myogenesis could be predicted for other miRNAs in this list because they regulate cellular proliferation (such as miR-132, miR-145 or miR-224), as well as cardiac hypertrophy (miR-378). Interestingly, the majority of these miRNAs were not found in the original loss-of-function screen, most likely due to the redundancy of miRNA family members. Indeed, as the miRNAs of the same family share the seed sequence, an efficient loss-of-function screen should have contained not only individual miRNA inhibitors, but also the inactivators of whole miRNA families, in order to avoid false negative results. In this sense, our analysis of these data has been important in supplementing a group of miRNA targets that could have been overlooked. This possibility is highlighted by the presence of known myogenesis regulatory miRNAs (miR-1, miR-216a) in the list resulting from the analysis, whereas they have not been picked up by the original experimental screen.

**Fig. 1:**
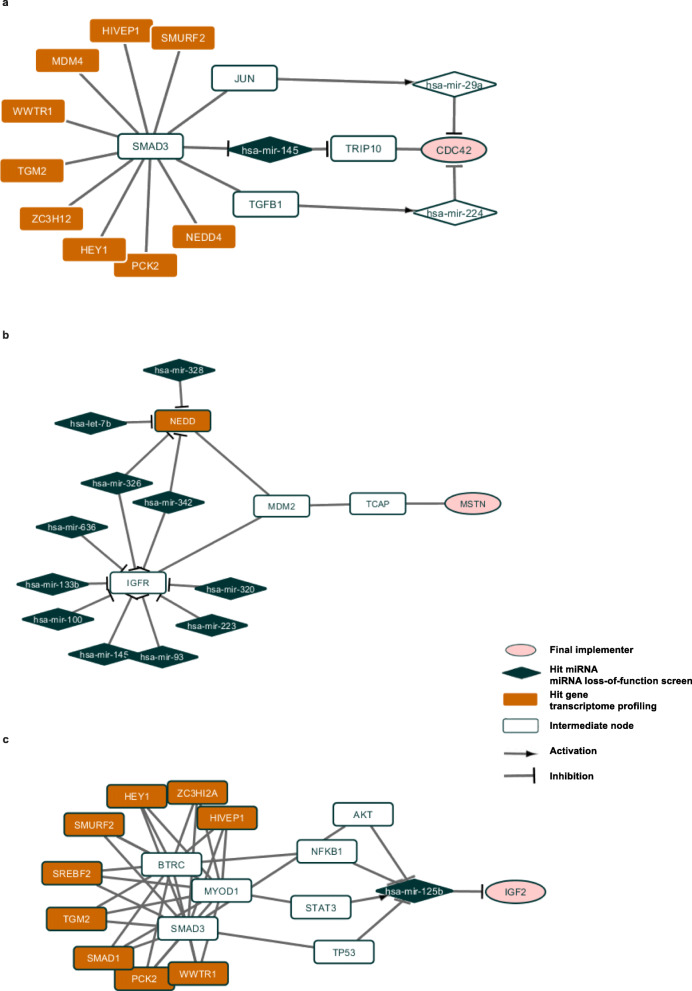
Subnetworks for human muscle differentiation process. Hit genes in miRNA loss-of-function screen are in dark blue, hit genes in transcriptome profiling are in orange, final implementers are in pink, intermediate genes and proteins are in white. **a** SMAD3-hsa-mir-145 subnetwork. **b** SMAD3, MYOD1 subnetwork. **c** MDM2-TCAP subnetwork

The analysis of paths allowed identification of potentially novel pathway in regulation of myogenesis, the clathrin-coated pathway regulatory protein AP2M1, and the EH-domain protein EHD2, which links the clathrin coated transport to actin cytoskeleton, and also binds to myoferlin, a factor promoting myotube fusion. Together with integrin subunits ITGA4 and ITGB1, the extracellular matrix component fibronectin (FN1), and the protein chaperon HSP90, these proteins indicate a possible involvement of specific protein transport pathways in terminal myogenic differentiation. In addition, there is a possibility of involvement of beta-catenin (CTNNB1), C-KIT and PRKC in these processes. It should be noted that these three regulatory factors, while extensively studied in a multitude of biological models, have never been shown to be specifically implicated in skeletal myogenesis. Two major regulatory molecules of skeletal myogenesis, MYOD and SMAD3 (Fig. 1 a, b), have been highlighted, together with their previously known muscle-related targets (TGFB1, CDC42, CTCF). Also, they are linked to such proteins as C-KIT, that have not been previously studied in the context of muscle differentiation.

Next, we compared the lists of nodes and the lists of paths from two experiments. We found 37 nodes and 20 paths common for both loss-of-function screening and transcriptome profiling. Among the nodes with the highest centrality score, two – IGF1R, E2F1 – have been suggested to play key roles in the growth, development, and differentiation of skeletal muscle [44–46]. The path with the highest score consists of CDKN1A, MDM2, TCAP, MSTN proteins. The interaction between MDM2 and TCAP is known to be important for cardiac hypertrophy [47], it was also shown that TCAP controls secretion of MSTN [48]. Our analysis shows that this path might be activated by the depletion of hsa-mir-17, hsa-mir-106a, hsa-mir-125a, hsa-mir-145, hsa-mir-93 (Fig. 1c). It can also be noted that not only androgen receptor (AR), but also the estrogen receptor ESR1 can play a role in human skeletal myogenesis. Interestingly, specific integrins (ITGB1) and adaptor proteins (CRKL) have also been found, confirming the importance of certain membrane/adherence structures. Strikingly, both the receptor of activated C kinase (RACK1), and the inhibitor of this kinase (YWHAB, a 14–3-3 protein), as well as multiple other protein-processing enzymes (casein kinase CSNK1A, activator or protein secretion CHRM3) were found by the analysis, attracting the attention to the role of protein metabolism in myogenesis. It was also very interesting to see the chromosome breakpoint generation factor FRA11B among these potential novel factors that might impact on the differentiation of human myoblasts. This comparison has shown potentially novel paths originating from well-known actors in muscle differentiation (such as IGF1R - RACK1 - CD81); and vice versa, has shown that previously unknown potential regulators of myogenesis, such as YWHAB or FRA11B, can act upon proteins that are well known to regulate myotube hypertrophy and/or fusion (IGF1R, CD81).

The fact that the comparison resulted only in a few number of paths might indicate, that although these experimental systems study one biological process, they characterize the biological machinery at two different levels: transcriptional (transcriptome) and post-transcriptional (miRNAs).

We also found that 22 paths from the analysis of transcriptome profiling have miRNAs hits from the loss-of-function screening and 260 paths from the analysis of loss-of-function screening have hit genes from the transcriptome profiling on them. These are the paths from the analysis of transcriptome profiling that pass through hsa-mir-125b which controls IGF2 gene and the paths that pass through hsa-mir-145 that control TRIP10 protein which, according to OMIM database, has highest expression in skeletal muscle [49] and interacts with CDC42 protein. Also, when analyzing these paths, one can notice the factors participating in at least three major cellular pathways, that, however, have not been extensively studied in skeletal muscle differentiation. These factors include beta-transduction repeat containing protein (BTRC), which has a strong impact on both beta-catenin and NF-kappa B signaling, as well as the p53-related protein TP73, and, finally, the protein LRIG1 that has a strong negative effect on the expression of epidermal growth factor receptor. These pathways represent promising new directions to follow in order to further understand the mechanics of skeletal myogenesis.

### Gene loss-of-function screen to identify genes regulating human oxidative DNA damage recognition

OGG1 is a DNA glycosylase that initiates the repair of 8-oxoG, a major base modification induced by oxidative stress. The induction of 8-oxoG results in the recruitment of OGG1 and the subsequent enzymes of the Base Excision Repair (BER) pathway to chromatin to perform repair [50–52]. A “druggable” loss of function siRNA screening (only genes from druggable part of the genome were targeted), using 3 independent siRNAs per gene, was performed on genetically engineered HeLa cells that stably express OGG1-GFP fusion protein [53–55]. The intensity of chromatin-bound OGG1-GFP was measured after inducing DNA damage. 18 of the obtained hit genes (Table S3) for which inactivation led to an impairment of recruitment were selected for further analysis.

All 18 hit genes were used as a list of final implementers, since little is known about the proteins involved in the recruitment of OGG1 to chromatin. The analysis was performed in the protein-protein human interactome.

We identified 4876 shortest protein-protein paths (from 1 to 4 interactions) going from each gene product in the hit list to each gene product in the list of final implementers. The subnetwork constructed from these paths consists of 381 nodes and 1764 edges without duplicated edges. The centrality score and the *p*-value were calculated for each node and path in the subnetwork according to the procedure described in the *Method* section. 396 paths with centrality score 3 of length of 3 interactions and no nodes were found at the FDR of 0.25.

Although no nodes were found at the selected FDR, we examined the full list of nodes to see whether nodes with high centrality were shown to be associated with DNA damage recognition process. Indeed, among nodes with the highest centrality score are ten H3 proteins: HIST1H3A, HIST1H3B, HIST1H3C, HIST1H3D, HIST1H3E, HIST1H3F, HIST1H3G, HIST1H3H, HIST1H3I, HIST1H3J. It is known that DNA damage is associated with higher level of chromatin mobility [56–58] and it was shown recently that the increase in chromatin mobility is governed by the proteasome-mediated degradation of core histones [59]. Other proteins with high centrality score are SETDB1 – a member of the SET1 family of proteins; WDR5 – a core component of SET1 family complexes [60]; TP53BP1 – a binding partner of the tumour suppressor protein p53. SETDB1 and WDR5 are associated with post-translational histone modifications which allow recruitment of the chromatin-associated proteins and protein complexes [61, 62]. TP53BP1 protein is known to be an important regulator of the cellular response to DNA double-strand breaks [63]. The reason why none of these nodes survived multiple testing by FDR can be explained by the specific choice of the hit list used in the analysis. This means that many genes important for this biological system (OGG1 driven human oxidative DNA damage recognition process) were not targeted in the screening, while they can be present in the randomly generated hit lists thus yielding higher centrality scores for these nodes and increasing their *p*-values. As it shown below, this does not prevent the paths that were found at the selected FDR to pass through some of these nodes.

**Fig. 2:**
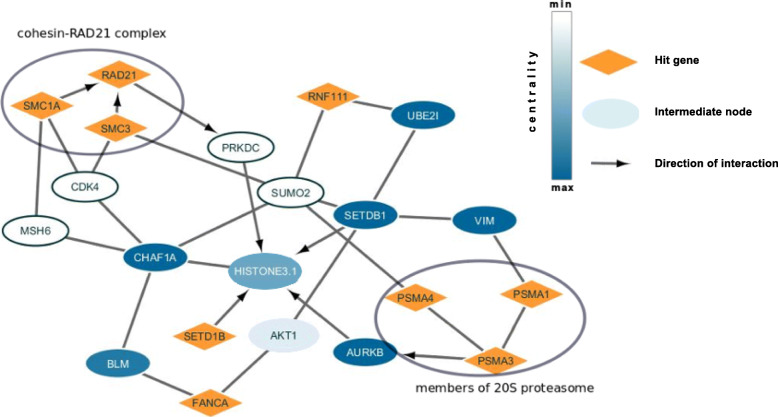
Histones H3-SETDB1 subnetwork for oxidative DNA damage recognition screening. Hit genes are in orange, the intermediate proteins are in white or blue depending on the centrality score. Grey arrows show the direction of interactions that were taken from literature

We examined the list of paths that were found at the selected FDR. Figure 2 presents a subnetwork visualized with Cytoscape software [64] for paths with centrality score 3. Figure 2 shows that the method identified two cohesin proteins SMC3 and SMC1A that interact with RAD21 protein to form cohesin-RAD21 complex [65], known to be enriched at DNA double-strand break sites and facilitating recombinational DNA repair [66]. It also shows possible mechanism of involvement of PSMA1, PSMA3, PSMA4 proteins, all members of the 20S proteasome [67], through interaction with AURKB, Aurora Kinase B [68], which in turn interacts with histones H3 [69]. The path ends with histones H3 – SETDB1 interaction. SETDB1 is a histone methyltransferase that specifically methylates histone H3 [61] and is also a member of the hit list. The arrows show the direction of the interactions inferred from literature. Considering them, histones H3 are the proteins where the signal from different members of the hit list converges and we hypothesize that histones H3 can be final implementers for this system.

### Implementation

MasterPath is available as a docker container. The usage instructions are presented in the Supplementary note 1. The Java source code and full tables of the results presented in the paper are available at the GitHub page https://github.com/daggoo/masterPath.

## Discussion

We used two different types of networks in our work. The first one was mixed directed and undirected network constructed from PPI, transcriptional, post-transcriptional and metabolic data. The second network was undirected PPI network. PPI networks are the most common networks used in network analysis, although they are known to be incomplete and biased towards the well-studied proteins. Incorporating transcriptional, post-transcriptional and metabolic data does not solve the issues associated with PPI networks, but adds information on direction, positive or negative effect of interactions and gives the ability to build heterogeneous paths.

The bias towards highly connected nodes or paths in the results that pass through highly connected nodes is controlled by generating random hit lists and performing multiple testing correction. As in Gene Set Enrichment Analysis [70], we used FDR threshold of 0.25 for predicted paths and nodes. We consider it an appropriate FDR threshold for an exploratory analysis which aims at generating new hypothesis for further validation. Also, nodes (respective paths) with higher *p*-values can be considered. However, in this case, the value of a centrality score and corresponding p-value can reflect not only specificity of a node (respective a path) for a biological process but can also be biased by high connectivity or incomplete hit gene list.

We used the following parameters for the analysis presented in this work. For the integrated network: maximum path length to search for the shortest paths between hit genes and final implementers (*L*_*max*_*, Methods* section) was 5 interactions, the paths of length 3 to 4 interactions were examined, minimum centrality score was 3 for both paths and nodes. For the protein-protein interaction network: *L*_*max,*_ was 4 interactions, the paths of length 2 to 3 interactions were examined, minimum centrality score was 3 for both paths and nodes. *L*_*max,*_ was chosen to approximate the average length of the shortest path in the networks (Table S4). We aimed at searching for the longest paths and considered paths one to two interactions shorter than *L*_*max*_. The use of the threshold for the centrality score of 3 can help to capture those situations when the hit list and the list of final implementers contain a small subset of elements that are connected only with each other in the network and thus cannot produce high centrality scores. It should be noted that these parameters can be changed based on the topology of the network in use.

Other important problems in network analysis are network specificity for the biological system of interest and lack of interaction information about certain members of a hit list. We used networks that represent global human interactome with high-confidence experimentally validate interactions in our work. Nodes that are not present in a particular interactome (e. g. tissue specific interactome) can be excluded from the network, based e. g. on transcriptomic data, to create smaller but more specific networks. On the other hand, it is typical to have poorly studied hit genes in hit lists from functional screens that might not be present in the network. Low confidence or predicted interactions for such hit genes might be added to the network in this case, which is especially important for interaction types other than protein-protein interactions.

Most of the protein-protein interactions in our global integrated network come from high confidence protein-protein interactions in HIPPIE database. HIPPIE contains interactions from widely used BioGRID [71], DIP [72], HPRD [73], IntAct [74], MINT [75] databases. All interactions in HIPPIE database have a confidence score which is calculated as a weighted sum of several parameters including number of studies in which an interaction was detected and the type of the experimental technique used to detect the interaction. The confidence score allows to filter out low confidence interactions. High confidence interactions from other databases (e. g. String [76]) or separate studies might be added to the network to improve network completeness and/or specificity for a particular biological system.

We introduced a notion of final implementer. We denoted a final implementer as a molecule that is involved in events responsible for the development of the final realization of the phenotype in the biological process of interest. Modern molecular biology accumulated vast amount of knowledge and such molecules are known for some processes, e. g. caspase 3, caspase 6 and caspase 7 could be considered as final implementers for apoptosis. If these molecules are unknown, the members of the hit list can be used as a list of final implementers in the analysis on the PPI network. In this case, candidates for final implementers could be found by studying direction of the paths, as we demonstrated for human oxidative DNA damage recognition process.

## Conclusion

We presented a new exploratory network analysis method that employs the shortest path approach and centrality measure to uncover members of active molecular pathways leading to a given phenotype, based on the results of functional screening. We illustrated the application of the method to the analysis of the results of transcriptome profiling and miRNA loss-of-function screening of human skeletal muscle differentiation process and of “druggable” loss-of-function screening of human DNA repair process.

## Methods

### Databases

The human integrated interactome was constructed from 7 databases: Human Integrated Protein-Protein Interaction rEference (HIPPIE) [77], SIGNOR [78], SignaLink [79], TFactS [80], KEGG Metabolic Pathways [81], TransmiR [82], miRTarBase [83].

All databases contain experimentally validated interactions, except SignaLink database which contains a small number of predicted miRNA-mRNA interactions. We used only high confidence interactions from HIPPIE database. The confidence threshold was chosen according to HIPPIE documentation [84]. Since all the databases use different types of gene ID, we converted the ids to the HUGO gene nomenclature and used this nomenclature to construct human integrated network. SIGNOR database contains some interactions that involve phenotypes, protein families and stimuli; however, we used only interactions between proteins, complexes and small molecules. Table 1 summarizes the basic information about the databases. Table S4 summarizes topological features of the integrated networks used in this work: integrated interactome constructed from all databases in Table 1 and undirected protein-protein interactome constructed only from HIPPIE database in Table 1.

**Table 1:** Number of nodes, interactions and types of interactions in databases used to construct human integrated network. PPI: protein-protein interactions, TF: transcription factor

Database	Nodes	Interactions	Types of nodes	Types of interactions	Direction of interactions
HIPPIE (high confidence)	9368	41,520	proteins	PPI	undirected
SIGNOR	3977	13,129	proteins, complexes, small molecules	PPI, enzymatic	directed, undirected
SignaLink	3285	27,295	proteins, genes, miRNAs	PPI, miRNA-mRNA, TF-gene	directed, undirected
TFactS	2203	4312	TFs, genes	TF-gene	directed
KEGG metabolic pathways	2921	8231	proteins, small molecules	Enzymatic reactions	directed
TransmiR	324	647	TFs, miRNAs	TF-miRNA	directed
miRTarBase	2269	3511	miRNAs, genes	miRNA-mRNA	directed

**Fig. 3:**
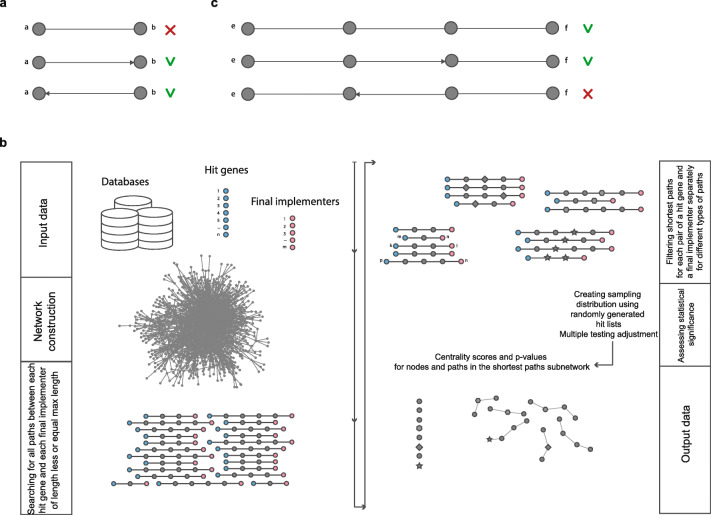
**a** Undirected interactions are not included into the integrated network during merging of databases in case directed interactions exist between the same nodes. **b** Main steps of the MasterPath. Detailed description is presented in the Method section. **c** Direction of interactions is taken into account when paths are found using breadth-first algorithm. Only the first two paths will be considered between nodes *e* and *f* by the method. It should be noted that the arrow represents here only the direction of the interaction but not the effect (e.g. activation or inhibition)

Integrated interactome contains both directed and undirected interactions. When the databases are merged to create the integrated network, duplicate interactions are removed. If there are both directed and undirected interaction between any two nodes, only directed interactions are kept in the integrated network (Fig. 3a).

### MasterPATH algorithm

The following notions are used in the mechanistic model of pathway construction. An unweighted graph *G = (V, E)* represents a network of molecular interactions. *V* are nodes that can be proteins, genes, small molecules or miRNAs. *E* are edges that represent molecular interaction between the nodes. The interactions can be directed or undirected. The list of the hit genes is a subset of nodes *H* from *V.* The list of final implementers is a subset of nodes *F* from *V*. A simple linear path *p* in the graph *G* between a pair of nodes *(v, u)* is a sequence of edges that connect *v* and *u: p(v, u)* = *(v, v*_*1*_*), (v*_*1*_*, v*_*2*_*) ... (v*_*k*_*, u)* where each *v*_*i*_ ∈ *V* and all *v*_*i*_ are distinct from one another. Length *L* of the path *p(v, u)* is the number of edges in the path*: p(v, u) = k + 1*.

We distinguish 4 different types of paths:

1. protein-protein paths if all edges in the path represent protein-protein interactions;
2. transcriptional paths if there exist at least one edge in the path that represent transcriptional interaction and all other edges represent protein-protein interactions;
3. miRNA paths if there exist at least one edge in the path that represent miRNA-mRNA interaction and all other edges represent either protein-protein or transcriptional interactions;
4. metabolic paths if there exist at least one edge in the path that represent enzymatic reaction and all other edges represent either protein-protein or transcriptional interactions or miRNA-mRNA interactions.

The algorithm of the method is the following (summarized in Fig. 3b). For a given network *G*, hit list *H*, list of final implementers *F* the method finds for each pair of a hit gene and a final implementer *(h*_*i,*_ *f*_*j*_*)* all the shortest paths *{p*_*i*_*}* of four mentioned types of length less or equal the maximum length *L*_*max*_ (defined by the user) in the network *G*. The search is done using breadth-first algorithm. The direction of the interactions but not the type of the interactions is taken into account during the search (Fig. 3c). Then the centrality score is calculated for each node *v* as the number of the shortest paths *{p}* from all combinations of hit genes and final implementers that pass through the node *v*: *c(v)* = | *p* ∈ *{p}*: *v* ∈ *p* |. The centrality score is calculated for each path *q* of length of several interactions as the number of the shortest paths *{p}* from all combinations of hit genes and final implementers for which *q* is a subpath: *c(q)* = | *p* ∈ *{p*_*i*_*}*: *q* is a subpath of *p* |. Centrality score *c(q)* for path *q* is taken as 1 if all the paths that have q as a subpath are of the same type and between the same combination of a hit gene and a final implementer to discriminate paths that pass through highly connected nodes. After that, the statistical significance of the centrality scores is assessed. 10,000 random hit lists are sampled from the set of nodes in the network preserving or not preserving the degree distribution of the initial hit list. The probability (p-value^Net^) of getting a node *v* or a path *q* with specific centrality score by chance is calculated as a proportion of sampled hit lists for which a node or a short path has the same or greater centrality score. Next, p-values^Net^ are adjusted for multiple testing using Benjamini-Hochberg procedure [85].

## Supplementary information

**Additional file 1: Supplementary note 1.** **Table S1.** Hit genes for miRNA loss-of-function screen of human muscle differentiation process. **Table S2.** Hit genes for transcriptome profiling of human muscle differentiation process. **Table S3.** Hit genes for human oxidative DNA damage recognition loss-of-function screen. **Table S4.** Topological features of the integrated and PPI networks.

## Data Availability

The datasets generated during current study as well as the source code are available at the GitHub page https://github.com/daggoo/masterPath. The datasets analyzed during current study are included in this published article’s supplementary information files and are available as a Supplementary File S1 in the reference publication [30], 10.1371/journal.pone.0071927.s004 and a Supplementary Table 1 in the reference publication [55], https://academic.oup.com/nar/advance-article/doi/10.1093/nar/gkaa611/5876283#supplementary-data

## Abbreviations

FDR: False discovery rate
HIPPIE: Human integrated protein-protein interaction reference database
HUGO: Human Genome Organisation
Id: Identifier
KEGG: Kyoto encyclopedia of genes and genomes
LHCN: Late differentiating human muscle precursor cell line
miRNA: microRNA
miRTarBase: database of microRNA - target interactions
mRNA: Messenger RNA
ORA: Over representation analysis
PPI: Protein–protein interaction
SIGNOR: Signaling network open resource database
siRNA: Small interfering RNA
TF: Transcription factor
TFactS: Transcription factor activity database
TransmiR: Database for transcription factor - microRNA regulations
