# Supplementary material for: MasterPATH: network analysis of functional genomics screening data
Source: BMC Genomics. 2020 Sep 14;21:632. doi: 10.1186/s12864-020-07047-2 (PMC7491077; doi:10.1186/s12864-020-07047-2)
Supplement: Supplementary file 1 — Additional file 1: Supplementary note 1. Table S1. Hit genes for miRNA loss-of-function screen of human muscle differentiation process. Table S2. Hit genes for transcriptome profiling of human muscle differentiation process. Table S3. Hit genes for human oxidative DNA damage recognition loss-of-function screen. Table S4. Topological features of the integrated and PPI networks. [file 12864_2020_7047_MOESM1_ESM.docx]

**Supplementary note 1**

**Usage instructions**

**Hit genes and final implementers files**

**Test parameters**

**MasterPath output**

**Visualization with Cytoscape software**

**Table S1**

**Table S2**

**Table S3**

**Table S4**

**2**

**2**

**3**

**4**

**4**

**5**

**6**

**7**

**9**

**10**

**Supplementary note 1**

**Usage instructions**

MasterPath is available from the docker container from *https://hub.docker.com/repository/docker/nrubanova/masterpath*

To use MasterPath from the container it is required to sign up for Docker Hub and install Docker Desktop or Docker Engine – Community (if on Linux). Please see the instructions on [*https://docs.docker.com/docker-hub/*](https://docs.docker.com/docker-hub/) (Linux) or [*https://docs.docker.com/docker-for-windows/install/*](https://docs.docker.com/docker-for-windows/install/) (Windows) .

MasterPath can be run in two modes: *‘paths’* and *‘network’*. Mode *‘paths’* performs analysis described in the paper. Mode *‘network’* creates files to visualize subnetworks constructed from the selected shortest paths.

To run MasterPath analysis in mode *‘paths’* (-m paths) from the terminal please use the following command:

docker run -v **<Folder_path_with_input_data>**:/input -v **<Folder_path_for_output_data>**:/output -it --rm nrubanova/masterpath:latest -n **<ppi_or_integrated>** -m paths -fp /input/**<File_name_Final_implemeter>** -hl /input**/<File_name_Hit_List>** -minl **<Minimum_length_of_path>** -minc **<Min_centrality_for_nodes_and_paths>** -maxl **<Maximum_length_of_path>** -ml **<Max_length_of_paths_for_Breads_first_algorithm>** -p **<Prefix>** -r **<Number_of_random_interactions>** -t **<Number_of_threads>** -o /output

To run MasterPath to create files for network visualization in Cytoscape (-m network) from the terminal please use the following command:

docker run -v **<Folder_path_with_input_data>**:/input -v **<Folder_path_for_output_data>**:/output -it --rm nrubanova/masterpath:latest -m network -n **<ppi_or_integrated>** -fp /input/**<File_name_Final_implemeter>** -hl /input**/<File_name_Hit_List>** -l /input/**<File_name_with_paths_IDs>** -p **<Prefix>** -o /output

where parameters that should be specified by the user are in bold font.

The command line parameters are:

-fp,--finalimplementers <arg> Final implementers file name

-hl,--hitlist <arg> Hit list file name

-l,--pathlist <arg> File with paths IDs to create files for Cytoscape.

-m,--mode <arg> Mode: paths or network

-maxl,--maxpathlength <arg> Max length of paths to report

-minl,--minpathlength <arg> Min length of paths to report

-minc,--mincentrality <arg> Min centrality for nodes and paths to test

-ml,--maxlength <arg> Max length of paths for Breadth first algorithm

-n,--network <arg> Network type: ppi or integrated

-o,--output <arg> Output folder. Specified with -v parameter for docker container

-p,--prefix <arg> Prefix for paths' ids

-r,--randominteractions <arg> Number of random interactions

-t,--threads <arg> Number of threads

**Hit genes and final implementers files**

Hit genes and final implementers should be in tab delimited files. Each element in a separate line. The IDs are preceded by the symbols. ‘p’ before official gene symbol indicate a protein.

Example:

Hit list

ATP6V0E2 HGNC:21723

ATP8B1 HGNC:3706

ATXN7L1 HGNC:22210

B3GALT5 HGNC:920

BAIAP2L1 HGNC:21649

BASP1 HGNC:957

BBS12 HGNC:26648

BCAM HGNC:6722

hsa-mir-106a hsa-mir-106a

has-mir-17 hsa-mir-17

hsa-mir-1227 hsa-mir-1227

Final implementers

MSTN pHGNC:4223

IGF2 pHGNC:5466

ACTA1 pHGNC:129

MYH1 pHGNC:7567

MYLPF pHGNC:29824

**Test parameters**

To test MasterPath use:

-fp test

-hl test

-m paths

-n integrated

-minl 2

-maxl 3

-ml 4

-p TST

-r 10

**MasterPath output**

The two main output files from MasterPath in --mode paths are:

*masterPATH_<prefix>_paths_centrality_pvalues* file with paths information in a tab separated text file.

File format:

path id; path as a list of interaction ids (each interaction is separated by a tab); reserved field; centrality; hit gene-final implementer pairs that yield this path as a list of HGNC ids separated by semicolon; list of the shortest paths ids that yield this path separated by semicolon; reserved field; reserved field, HGNC id of the node in the path with maximum centrality; official symbol of the node in the path with maximum centrality; hit gene-final implementer pairs that yield this path as a list of official symbols separated by semicolon; path as a list of intercator1-interactor2 pairs (each interaction is separated by a tab); centrality; p-value; adjusted p-value.

*masterPATH_<prefix>*_*nodes_centrality_pvalues* file with nodes information in a tab separated text file.

File format:

node id; node ofiicial symbol; centrality; p-value; adjusted p-value.

**Visualization with Cytoscape software**

Using MasterPath with --mode network parameter allows creating files for network visualization with Cytoscape software. Each line of the resulting file with centrality scores for paths contains Column 5 with the list of IDs of the shortest paths that yielded this particular path. These IDs can be copied into a separate file *<file name>* where one ID is placed on one line (for example, for a test run:

TST1

TST20

TST23

) and the file can be specified with -l parameter when masterPath is run with --mode network parameter. This will produce two files that can be loaded into Cytoscape:

*<file name>_interactions_cyto* contains lines each representing an interaction. The file can be loaded into Cytoscape via *File > Import > Network from file* to create a network.

Column 1 is ID of the first interactor

Column 2 is ID of the second interactor

Column 3 is type of the interaction

Column 4 shows whether interaction is directed or undirected

*<file name>_nodes_cyto* contains nodes’ attributes (official symbols and type). It can be loaded to Cytoscape after creating the network via *File > Import > Table from file*.

**Table S1.** Hit genes for miRNA loss-of-function screen of human muscle differentiation process.

| hsa-mir-100 |
| --- |
| hsa-mir-106a |
| hsa-mir-17 |
| hsa-mir-1227 |
| hsa-mir-1233 |
| hsa-mir-125b |
| hsa-mir-1267 |
| hsa-mir-130b |
| hsa-mir-138-1 |
| hsa-mir-145 |
| hsa-mir-1538 |
| hsa-mir-18b |
| hsa-mir-223 |
| hsa-mir-296 |
| hsa-mir-326 |
| hsa-mir-331 |
| hsa-mir-339 |
| hsa-mir-365 |
| hsa-mir-429 |
| hsa-mir-454 |
| hsa-mir-455 |
| hsa-mir-484 |
| hsa-mir-485 |
| hsa-mir-501 |
| hsa-mir-512 |
| hsa-mir-532 |
| hsa-mir-541 |
| hsa-mir-600 |
| hsa-mir-625 |
| hsa-mir-636 |
| hsa-mir-663 |
| hsa-mir-664 |
| hsa-mir-766 |
| hsa-mir-770 |
| hsa-mir-93 |
| hsa-let-7b |
| hsa-mir-1224 |
| hsa-mir-1228 |
| hsa-mir-1249 |
| hsa-mir-125a |
| hsa-mir-1260 |
| hsa-mir-1280 |
| hsa-mir-129 |
| hsa-mir-1296 |
| hsa-mir-133a |
| hsa-mir-133b |
| hsa-mir-150 |
| hsa-mir-197 |
| hsa-mir-204 |
| hsa-mir-328 |
| hsa-mir-342 |
| hsa-mir-346 |
| hsa-mir-361 |
| hsa-mir-483 |
| hsa-mir-486 |
| hsa-mir-574 |
| hsa-mir-629 |
| hsa-mir-885 |
| hsa-mir-193b |
| hsa-mir-369 |
| hsa-mir-381 |
| hsa-mir-886 |
| hsa-mir-940 |
| hsa-mir-98 |
| hsa-mir-631 |

**Table S2.** Hit genes for transcriptome profiling of human muscle differentiation process.

AASS

ABCA1

ABCC3

ABHD5

ABLIM3

ACCN2

ACOT11

ACTA1

ADAM9

ADAMTS6

ADAMTSL1

ADAMTSL5

AFAP1L1

AFAP1L2

AGPAT5

AGRN

AIF1L

AIM1

AKAP1

ALCAM

AMIGO2

ANK2

ANKRD10

ANKRD13A

ANKS1A

APOBEC2

APOBEC3C

APOC1

APOD

APOE

AQP3

ARHGAP9

ARHGEF2

ARHGEF3

ARHGEF6

ARPC1B

ARPP21

ASNS

ASPM

ASS1

ASTN2

ATOH8

ATP10A

ATP1A1

ATP6V0D1

ATP6V0E2

ATP8B1

ATXN7L1

B3GALT5

BAIAP2L1

BASP1

BBS12

BCAM

BCHE

BCL2L11

BCL6

BCL7A

BDKRB1

BEND7

BHLHE41

BIN1

BIRC5

BLCAP

BNC1

BNIP3

BTG1

BTG3

C10orf10

C10orf72

C11orf71

C12orf34

C14orf159

C14orf43

C1S

C1orf105

C2

C20orf103

C21orf119

C21orf33

C21orf59

C21orf88

C22orf13

C3

C3orf39

C4A

C7orf55

C7orf58

C9orf46

CABLES1

CACNA1S

CACNA2D1

CACNB1

CACNG1

CAMK2D

CASP9

CASQ2

CASS4

CASZ1

CBFB

CCDC102B

CCDC99

CCNA2

CCND3

CD36

CD68

CDC25B

CDH2

CDK1

CDK15

CDKN1C

CDKN3

CDON

CELA2B

CENPA

CENPE

CGB

CGB1

CGB2

CGB5

CGB7

CHCHD8

CHD7

CHIT1

CHRNB1

CHRND

CHRNG

CIAO1

CKB

CKM

CLCA2

CLCN5

CLSTN2

COL5A3

COL6A2

COL6A3

CORO2B

CORO7

CPA4

CPNE2

CPS1

CREB3L1

CREG1

CRIM1

CTNNAL1

CTSH

CUEDC1

CUZD1

CYB5R1

DAAM2

DAG1

DBH

DBNDD1

DCBLD1

DCP2

DDI2

DGKD

DIAPH3

DIO2

DLG2

DLL1

DMBT1

DNAJB1

DNASE1L1

DOCK10

DOCK11

DOCK5

DPT

DSP

DTNA

EAF1

EBF1

ECH1

ECM1

EDN1

EFHD2

EGLN3

ELN

ENO3

ENPP1

ENPP2

EPHB2

ERBB3

ERO1L

EZR

FAIM2

FAM101B

FAM171A2

FAM174A

FAM65B

FBN1

FBXL22

FGF1

FGF2

FGFR4

FGL2

FILIP1

FITM1

FLVCR2

FMN2

FMNL1

FMO3

FNDC5

FOXM1

FRK

FRMPD1

FSTL3

FXYD1

FXYD5

FXYD6

FYCO1

G6PD

GAB2

GADD45G

GALNT5

GATM

GCNT1

GCOM1

GGH

GGT5

GLIPR1

GLRX

GMPR

GNAZ

GPM6B

GPNMB

GPR1

GPR125

GPR153

GPR155

GPR176

GREM1

GSTT2

GTF2F2

GUSB

H1F0

HES1

HES6

HEY1

HFE2

HIP1

HIPK3

HIST1H3J

HIST2H4A

HIVEP1

HOMER2

HOXA7

HRC

HSD11B2

HSD17B10

HSPA4L

HSPB8

ID1

ID2

ID3

IFFO1

IGF1

IGFBP6

IL1R1

IL34

IMPA2

IMPAD1

INHBA

INPP4B

INPPL1

ITGA10

ITGA7

ITGB1BP2

ITGB1BP3

ITM2C

KCNA7

KCNIP3

KCNJ15

KCNJ2

KCNN3

KCNQ4

KDELR3

KDM4B

KDM6B

KIAA1632

KIAA1644

KIF13B

KIF1B

KIF20A

KLHL28

KREMEN1

KY

LAMA4

LAMA5

LAPTM5

LDB3

LDLRAD3

LETMD1

LMBRD2

LMOD3

LNX1

LOC645166

LOC654342

LOX

LRIG1

LRRC16A

LRRC17

LRRFIP1

LRRN1

LRRN4CL

LSP1

LSS

MACF1

MACROD1

MAMLD1

MAN2A2

MAP3K14

MAPRE3

MDM4

MEF2D

MEGF10

MEOX1

MFAP4

MFAP5

MGC39372

MICAL1

MICAL2

MIR133B

MIR206

MIR503

MLLT6

MOAP1

MOBKL1A

MORC4

MPI

MPP6

MRAP2

MRC2

MT1E

MTSS1

MTSS1L

MUSK

MYBPC2

MYBPH

MYF5

MYH3

MYH8

MYL4

MYL5

MYL6B

MYLPF

MYO18B

MYO5A

MYOG

MYOZ2

NCAPG

NCOA1

NDC80

NDRG2

NDRG4

NEB

NEDD4

NES

NEU1

NIPAL3

NOTCH3

NRG1

NRP2

NTM

NUDT14

NUP93

OLFM2

OLFML2A

OPHN1

OSR2

OTOF

OTUD3

OXTR

P2RX6

PAAF1

PACS2

PADI2

PAN2

PAR5

PAWR

PCBD1

PCK2

PDE2A

PDGFC

PDK1

PDLIM1

PFKM

PGA3

PGA4

PGA5

PGAM2

PGBD5

PGD

PGM2

PGM3

PID1

PLAC9

PLAU

PLEKHA4

PLOD2

PLXNA1

PLXNA2

PODN

PODXL

PORCN

PPFIA4

PRICKLE2

PRKAG3

PRKAR1A

PRKX

PRUNE

PSEN2

PSG4

PTGDS

PTGFR

PTGS1

PTN

PTPRF

PTTG1/PTTG1

PURB

PUS7L

RAB15

RAB3B

RAI14

RALA

RAPH1

RARRES3

RASA1

RASSF4

RBM24

RCL1

RCSD1

RDH5

RGR

RGS16

RGS2

RNASE3

ROS1

RPL39L

RPRD1B

RTN2

RYR1

SARS2

SBK2

SCARNA13

SCD

SCG2

SCGB1C1

SCN4A

SEMA6B

SEPT4

SEPW1

SERPINB8

SERPINE1

SERPINE2

SERPINF1

SETD7

SGOL1

SHD

SHF

SIM1

SIRT2

SIX4

SLC12A2

SLC12A7

SLC29A1

SLC38A3

SLC38A4

SLC38A6

SLC3A2

SLC43A2

SLC7A5

SLC7A7

SMAD1

SMPX

SMURF2

SMYD1

SNAI1

SNED1

SNORD113-4

SNORD116-17

SNORD116-20

SNORD116-3

SOX8

SPARCL1

SPOCK1

SPPL2B

SPRY1

SREBF1

SREBF2

SRF

SRGN

SRPK3

SSPO

SSX4

ST3GAL5

ST6GALNAC4

ST8SIA5

STAC3

STARD5

STAT3

STK10

SUN2

SYNPO

TAS1R1

TBC1D2

TBX15

TCEAL1

TCF12

TCN2

TDRKH

TEAD4

TES

TFCP2

TFF3

TGFBI

TGM2

THBD

THRA

TIMM8A

TIMP3

TMED4

TMEM119

TMEM169

TMEM171

TMEM205

TMEM25

TMEM38A

TMEM8C

TMOD1

TMTC1

TNFRSF11B

TNFRSF25

TNIK

TNNC2

TNNI1

TNNI2

TNNT3

TOP2A

TPCN1

TPM3

TPPP3

TRAPPC2L

TRIB2

TRIM62

TRIO

TSPAN14

TSPAN33

TTC13

TTN

TTYH2

UBE2G2

UBE2W

UCP2

UGDH

UNC119

UNC45B

UTS2R

VANGL1

VAX2

VEGFC

VGLL2

WDR5

WIPF1

WRNIP1

WWTR1

ZBTB7C

ZC3H12A

ZC3H8

ZNF238

ZNF512

ZNF778

**Table S3.** Hit genes for human oxidative DNA damage recognition loss-of-function screen.

| CHD4 |
| --- |
| DDB1 |
| DNM1 |
| FANCA |
| KIFC3 |
| MED12 |
| MED14 |
| PSMA1 |
| PSMA3 |
| PSMA4 |
| RAD21 |
| RBX1 |
| RNF111 |
| SETD1B |
| SMC1A |
| SMC3 |
| SMURF1 |
| UBE2B |

**Table S4.** Topological features of the integrated and PPI networks. The exponent of the fitted power-law distribution to the degree distribution was calculated with powerlaw [1] Python package.

|  | Integrated network | PPI network |
| --- | --- | --- |
| Nodes | 13 419 | 9 365 |
| Interactions | 85 277 | 41 329 |
| Average clustering coefficient | 0.11 | 0.15 |
| Number of connected components | 126 | 121 |
| Average number of neighbors | 9.2 | 8.6 |
| Density | 0.0007 | 0.0009 |
| Diameter | 25 | 12 |
| Average length of the shortest path | 4.5 | 4.1 |
| Exponent of fitted power-law distribution (total degree) | 3.16 | 2.74 |
| Exponent of fitted power-law distribution (in degree) | 3.93 | - |
| Exponent of fitted power-law distribution (out degree) | 2.36 | - |

1. Alstott, Jeff, Ed Bullmore, and Dietmar Plenz. 2014. “Powerlaw: A Python Package for Analysis of Heavy-Tailed Distributions.” *PLoS ONE* 9 (1). https://doi.org/10.1371/journal.pone.0085777.
